# The role of human extracellular matrix proteins in defining *Staphylococcus aureus* biofilm infections

**DOI:** 10.1093/femsre/fuae002

**Published:** 2024-02-09

**Authors:** Mohini Bhattacharya, Alexander R Horswill

**Affiliations:** Department of Immunology and Microbiology, University of Colorado School of Medicine, Aurora, CO 80045, United States; Department of Immunology and Microbiology, University of Colorado School of Medicine, Aurora, CO 80045, United States; Department of Veterans Affairs, Eastern Colorado Health Care System, Aurora, CO 80045, United States

**Keywords:** biofilm, MRSA, matrix, host, Infection, treatment

## Abstract

Twenty to forty one percent of the world’s population is either transiently or permanently colonized by the Gram-positive bacterium, *Staphylococcus aureus*. In 2017, the CDC designated methicillin-resistant *S. aureus* (MRSA) as a serious threat, reporting ∼300 000 cases of MRSA-associated hospitalizations annually, resulting in over 19 000 deaths, surpassing that of HIV in the USA. *S. aureus* is a proficient biofilm-forming organism that rapidly acquires resistance to antibiotics, most commonly methicillin (MRSA). This review focuses on a large group of (>30) *S. aureus* adhesins, either surface-associated or secreted that are designed to specifically bind to 15 or more of the proteins that form key components of the human extracellular matrix (hECM). Importantly, this includes hECM proteins that are pivotal to the homeostasis of almost every tissue environment [collagen (skin), proteoglycans (lung), hemoglobin (blood), elastin, laminin, fibrinogen, fibronectin, and fibrin (multiple organs)]. These adhesins offer *S. aureus* the potential to establish an infection in every sterile tissue niche. These infections often endure repeated immune onslaught, developing into chronic, biofilm-associated conditions that are tolerant to ∼1000 times the clinically prescribed dose of antibiotics. Depending on the infection and the immune response, this allows *S. aureus* to seamlessly transition from colonizer to pathogen by subtly manipulating the host against itself while providing the time and stealth that it requires to establish and persist as a biofilm. This is a comprehensive discussion of the interaction between *S. aureus* biofilms and the hECM. We provide particular focus on the role of these interactions in pathogenesis and, consequently, the clinical implications for the prevention and treatment of *S. aureus* biofilm infections.

## Teaser

A comprehensive discussion on the role of common host extracellular matrix proteins and how they contribute to biofilm formation during infections caused by the pathogen *Staphylococcus aureus*.

## Introduction

### Epidemiology and significance of *Staphylococcus aureus* biofilms to human health

According to an announcement made by the National Institutes of Health, upwards of 60% of all microbial infections are associated with biofilms (Lewis 2001). It is important to note that biofilms are not an exception but rather the predominant lifestyle in the microbial world (Costerton et al. 1987, Busscher and Van Der Mei 1995, Hall-Stoodley and Stoodley 2005, Lindsay and von Holy 2006, Ouidir et al. 2022). Biofilms are easily formed on both biotic and abiotic surfaces. Fifty to seventy percent of all hospital-acquired infections are estimated to originate from the introduction of medical devices. This includes the introduction of catheters, pacemakers, implants, and contact lenses, all of which can act as substrates for the development of *Staphylococcus aureus* biofilms (Darouiche 2004, Stoodley et al. 2008, Kathju et al. 2014, Jamal et al. 2018). Regardless of the point of entry, the biofilm lifecycle involves the dispersion of bacteria to secondary locations, resulting in severe infections. For example, drinking water contaminated with *Pseudomonas aeruginosa* can develop into pneumonia and cause severe lung damage or death (Berrouane et al. 2000, Maharaj et al. 2017, Fujiki et al. 2022). Similarly, the introduction of pacemakers, potentially contaminated by transiently colonizing bacteria or through transport and handling by colonized hospital personnel, allows *S. aureus* biofilms to form and disperse, resulting in difficult-to-treat cases of sepsis and endocarditis (Rali et al. 2019, Grapsa et al. 2022).

Although biofilm-forming bacteria are often isolated from hospitals, there is an underappreciation for this lifestyle, especially in the clinical management of disease (Lindsay and von Holy 2006). Biofilm infections are known to be up to 1000 times more recalcitrant to antimicrobial interventions, including treatment with antibiotics that are used successfully against planktonic bacteria, at the same or lower dosage (Ceri et al. 1999, Howlin et al. 2015). The failure of antimicrobials against biofilm infections is attributed to multiple biofilm-associated properties, including genetic alterations in the bacterium (Lewis 2001, 2010). This can cause the development of additional antibiotic-tolerant and resistant populations that confound the treatment of secondary infections.

The identification and treatment of biofilms is further complicated by the difficulty in isolating and culturing biofilm communities from patient samples. While biofilm biomass can often be isolated from the primary infection site (such as a catheter), traditional culturing techniques do not accurately capture bacterial burden, resulting in the incorrect estimation of antibiotic susceptibility and ultimately unsuccessful treatment of the infection (Høiby et al. 2015). Lastly, multiple studies confirm that once a biofilm is formed, the ability of the host immune system to eradicate the infection decreases exponentially, conversely increasing the possibility of persistent infection (Brinkmann et al. 2004, Berends et al. 2010, Bhattacharya et al. 2020).

### *Staphylococcus aureus* biofilm structure and general properties

The lifecycle of *S. aureus* biofilms can be broadly classified into four phases: attachment, proliferation, maturation, and dispersion. Biofilms are formed when bacteria attach to a surface, proliferate, and achieve sufficient population density to communicate with each other via chemical signals, in a process known as quorum sensing (Donlan and Costerton 2002, Stoodley et al. 2002). In *S. aureus*, this function is performed by the activities of the accessory gene regulator (Agr) system. Quorum sensing by the Agr system requires the expression of four proteins. While AgrC and AgrA are histidine kinase-response regulator partners, AgrB is a transmembrane protein that facilitates the transport of the autoinducing peptide encoded by *agrD* (Novick 2003). This circuit activates downstream genes that allow for the expression of proteins and polysaccharide intercellular adhesin (PIA), which, together with extracellular DNA, comprise the bacterial-derived components of the extracellular polymeric substance (EPS), allowing for maturation (Arciola et al. 2001, Boles et al. 2010, Speziale et al. 2014, Arciola et al. 2015).

**Figure 1.**
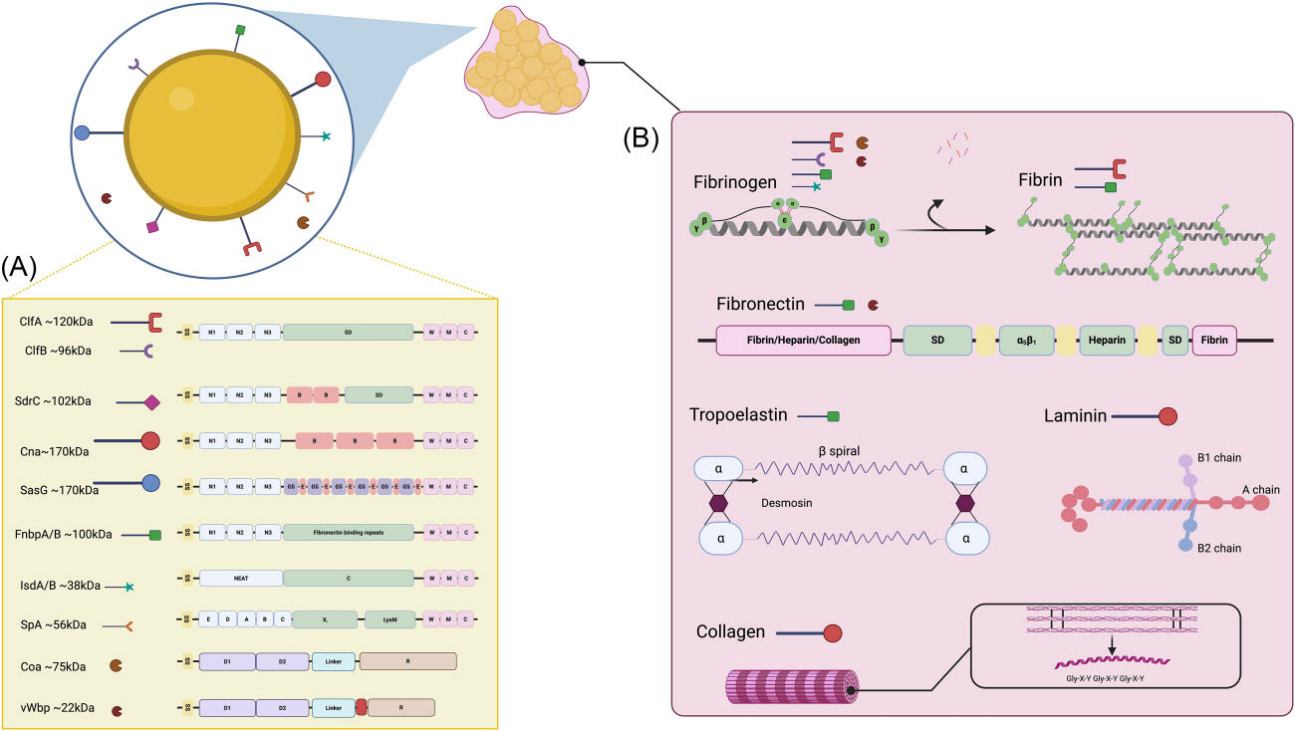
Proteins associated with *S. aureus* biofilms *in vivo*. Graphical representation providing a comparison of the functional motifs of surface and secreted proteins with significant contributions to *S. aureus* biofilm formation, as discussed in this review article. Symbols reflect protein size (provided in approximate kDa) (A). Depiction of the structures of 5 major hECM proteins that can be incorporated into the *S. aureus* biofilm EPS, as discussed in this review. *Staphylococcus aureus* surface and secreted proteins that are known to use each of these hECM proteins as ligands are shown in each panel. Proteins from A that are not depicted in B are known to have roles in inter-bacterial aggregation (B). Image was created with BioRender.com.

During the transition between each of the four phases of the biofilm lifecycle, the Agr regulatory network influences the expression of a majority of virulence factors made by the pathogen. This includes adhesins such as clumping factors A and B (ClfA, ClfB), fibrinogen-binding proteins A and B (Fnbp A, B), and others, which assist in the attachment to surfaces as well as the development of the biofilm EPS *in vivo* (Fig. 1A) (Novick 1991, 2003). Agr positively regulates the expression of virulence factors such as alpha hemolysin, gamma hemolysin, and Panton Valentine leucocidin, which have specific functions in allowing biofilm bacteria to evade macrophage and neutrophil-mediated killing (Scherr et al. 2015, Bhattacharya et al. 2018). During the dispersion of bacteria from the biofilm, Agr positively regulates the expression of phenol-soluble modulins (PSMs), a group of peptides that act as surfactants, as well as DNases and proteases that can cleave matrix components, further supporting the transition to planktonic life (Peschel and Otto 2013, Dastgheyb et al. 2015, Bhattacharya et al. 2018). In addition to the Agr network, there are a handful of global regulators and two-component systems that can play significant roles during biofilm development and pathogenesis. This includes at least 16 two-component systems that sense and respond to various environmental cues. The reader is referred to these comprehensive reviews for a better understanding of the regulatory networks that interconnect with the Agr quorum sensing circuit (Jenul and Horswill 2019, Crosby et al. 2020, Bleul et al. 2022).

**Figure 2.**
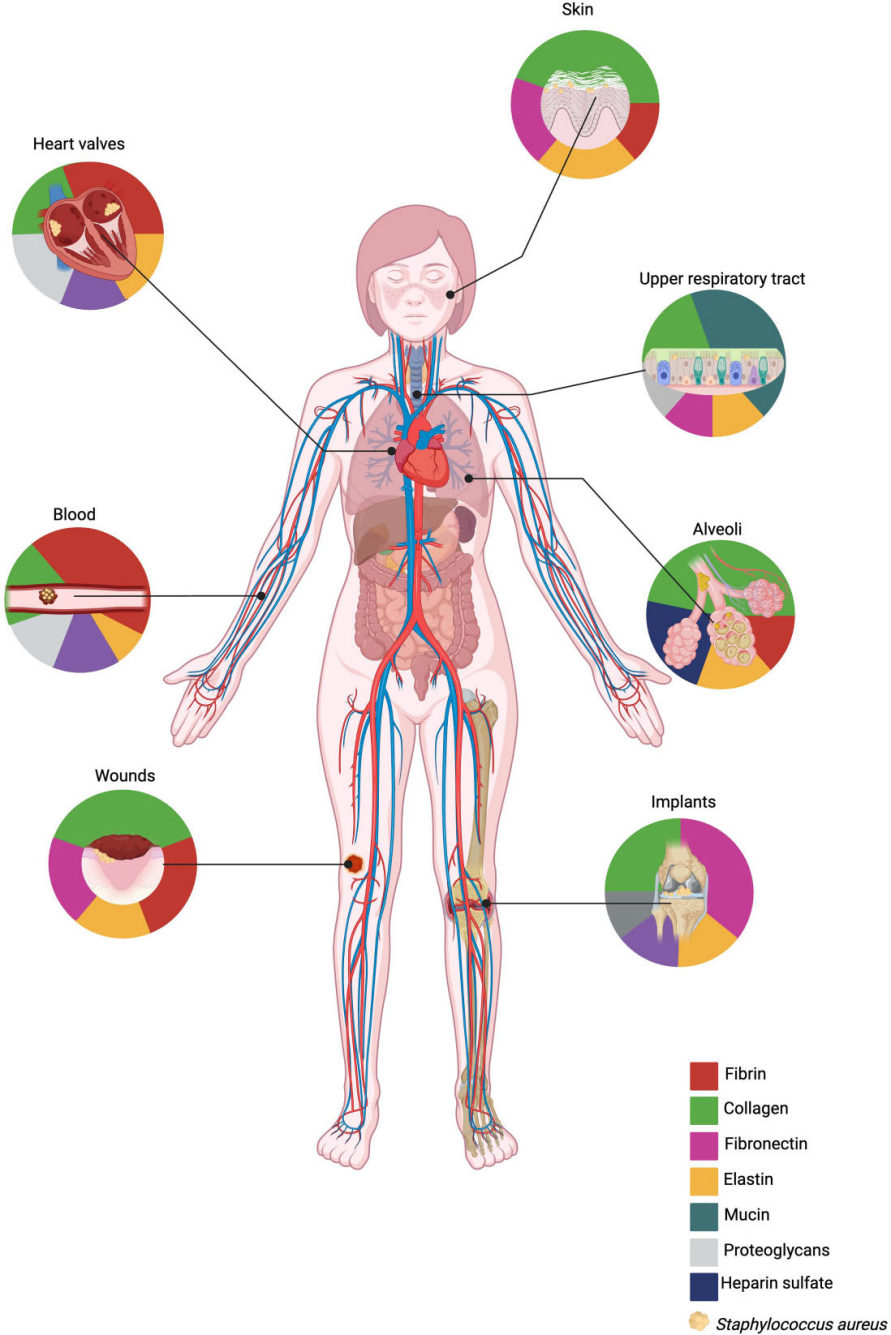
Human extracellular matrix differentially contributes to *S. aureus* biofilm infections. Diagrammatic summary of some key examples of biofilm-associated *S. aureus* infections as discussed in this review. Colors represent the relative, known contributions of various extracellular matrix-associated proteins (see legend) to *S. aureus* infections. The distribution of these colors is based on the known contributions of each hECM component during *S. aureus* biofilm-associated infections, to the extent that has been discussed in this manuscript. Distribution of colors in each chart is not intended to reflect a quantitative measurement. Image was created with BioRender.com

*Staphylococcus aureus* has evolved to be uniquely suited for survival in a human host. Unlike most bacterial pathogens, *S. aureus* can bind or utilize every major hECM protein, thereby causing a wide variety of infections with varied and specific pathogeneses (Howden et al. 2023). With a tight control over the transcription of virulence factors, *S. aureus* is able to quickly sense its environment and temporally express a signature of adhesins and secreted proteins required to survive in a particular tissue niche (Beenken et al. 2004). This review provides a comprehensive discussion of both partners in this interaction, i.e. the bacterium and the host. We therefore begin by elaborating on our current knowledge of factors that are expressed by *S. aureus* and allow it to bind and form biofilms with the hECM (Fig. 1A). Following this, we provide a detailed description of the major hECM components that potentiate *S. aureus* biofilm infections in the human host (Fig. 1B). We follow this with a discussion of some of the major biofilm-associated infections caused by *S. aureus*, with emphasis on the role of hECM components in each niche (Fig. 2). Lastly, this review briefly describes current and future avenues for anti-biofilm therapeutics that interfere specifically with these hECM-pathogen interactions.

### *Staphylococcus aureus* factors binding to the host matrix

#### Surface adhesins

*Staphylococcus aureus* strains can express at least 24 surface-anchored proteins that bind multiple known ligands from a single domain or multiple ligands from multiple domains of a single protein (Schneewind and Missiakas 2019). A large group of these surface proteins are crosslinked to the cell wall peptidoglycan via the activity of one or more of a group of sortase enzymes that can recognize the canonical LPXTG motif at the C-terminus of the protein (Fig. 1A). Ligand binding commonly occurs at the N-terminus of surface-anchored proteins; however, binding of host ligands to additional domains on the C-terminal end has also been reported (FnbpA, FnbpB) (Bingham et al. 2008). While the overall structure of these surface proteins is similar, they can be grouped into subclasses depending upon their functional domains (Sdr domain, NEAT domain proteins etc.). For the purposes of this review, a brief discussion of these proteins, categorized based on known function and potential relevance to biofilm formation, is provided here. For a more detailed analysis of the biochemistry and structure of these proteins, the reader is referred to the following expert reviews (Foster et al. 2013, Schneewind and Missiakas 2019).

*Fibronectin*-*binding proteins*. FnbpA and B are sortase-anchored proteins that consist of an N-terminal A domain that is currently characterized as binding to elastin, histones, plasminogen, and fibrinogen, followed by 10 (FnbpB) or 11 (FnbpA) fibronectin binding repeats at the C-terminus, each binding to the N-terminal domain of fibronectin with varying affinities using a β-zipper mechanism (Bingham et al. 2008, Pietrocola et al. 2016, Giampiero et al. 2019). While the A-domain is thought to promote interbacterial attachment and platelet aggregation, the C-terminus binds to fibronectin and often uses it as a bridge to interact with α_5_β_1_ integrin, a common cell surface receptor (Maurer et al. 2016).

One study performed with isolates from device-related infections detected FnbpA in 99% of *S. aureus* samples (Arciola et al. 2005). Indeed, FnbpA has been found to have particular importance in periprosthetic implant-associated infections caused by *S. aureus*. Studies with human serum and joint synovial fluid demonstrate that FnbpA is required for optimum biofilm formation and that it protects bacteria against phagocytic attack (Gries et al. 2020, Pestrak et al. 2020). While deletion of genes encoding for both proteins has been shown to significantly affect biofilm formation, eliminating the expression of either FnbpA or FnbpB individually has not resulted in substantial effects on the capacity of *S. aureus* to form biofilms, indicating an additive role for the two proteins during infection (O'Neill et al. 2008).

*Serine-aspartate-repeat (Sdr) domain proteins*. ClfA and ClfB are canonical members of a subgroup of cell wall-anchored proteins known as the Sdr family of adhesins, characterized by a string of ∼150 serine aspartate repeats at the C-terminus. This group consists of 5 proteins: ClfA, ClfB, SdrC, SdrD, and SdrE (Fig. 1A). The Sdr repeats characteristic of this group are glycosylated with N-acetyl glucosamine at every serine residue. The addition of this glycosyl group is attributed to the combined enzymatic activity of two glycosyl transferases, namely, AggB/SdgA and AggC/SdgB. While a role for this repeated glycosylation is yet to be appreciated, the potential contribution of this modification to interbacterial attachment and therefore aggregate biofilm formation has been postulated (Hazenbos et al. 2013, Thomer et al. 2014). While ClfA and ClfB bind to soluble fibrinogen with high affinities, ClfA can additionally bind insoluble fibrin (polymerized fibrinogen). ClfA is essential for the survival of *S. aureus* during bloodstream-associated infections, attributed to its ability to cause bacterial-fibrinogen interactions that result in agglutination of blood plasma, a form of aggregate biofilm formation that is characteristic to *S. aureus* (McAdow et al. 2011). Additionally, ClfA can indirectly engage with von Willebrand factor (vWf), annexin 2a, and the platelet receptor glycoprotein IIIb, allowing numerous interactions to occur both in blood and tissue, leading to bacterial accumulation and biofilm formation (Mcdevitt et al. 1997, Ashraf et al. 2017, Claes et al. 2017, Herman-Bausier et al. 2018). The utility of ClfA as a therapeutic target for *S. aureus* infections has resulted in multiple attempts at monoclonal vaccines targeting the adhesin (Ganesh et al. 2008, Tkaczyk et al. 2016). Indeed, recent studies demonstrate the efficacy of anti-ClfA antibodies in reducing the bacterial burdens of murine hematogenous implant-associated biofilms (Wang et al. 2017a,b).

ClfB plays a significant role in augmenting biofilm formation under conditions of calcium starvation in numerous strains of *S. aureus* (Abraham and Jefferson 2012). The role of ClfB as an important adhesion factor during the early attachment phase of biofilm development is evident from numerous studies that demonstrate the requirement of ClfB for the adhesion of bacteria to the nasal epithelium using murine models of colonization. This activity is attributed to the cytokeratin-10 binding capacity of the A-domain of ClfB (Schaffer et al. 2006, Wertheim et al. 2008, Sun et al. 2018). Another Sdr protein with noteworthy contributions to biofilm formation is SdrC. In addition to forming low-affinity homophilic bonds between neighboring cells, thereby facilitating bacterial accumulation, SdrC has also been shown to form hydrophobic bonds with abiotic surfaces, promoting the attachment of aggregates to a substrate, such as would be found during implant-associated infections (Barbu et al. 2014, Feuillie et al. 2017).

*Staphylococcus aureus surface (Sas) protein family*. In addition to the Sdr group of proteins, *S. aureus* strains can also express up to eight surface-associated (Sas) proteins, initially named due to their discovery in association with the cell wall. SasG is the best-characterized of these proteins, particularly for its ability to augment the formation of aggregate biofilms. With structural and functional homology to the accumulation-associated protein (Aap) of *S. epidermidis* as well as the plasmin-sensitive protein of *S. aureus* (Pls), SasG has a C-terminus comprised of multiple “B” domains containing 5–8 G5 subdomains (∼78 amino acids) separated by “E” subdomain linkers ∼50 amino acids long (Fig. 1A). The B-domain of SasG is heavily processed by proteases, resulting in the formation of both secreted B-domain peptides and surface-associated B-domains formed following the cleavage of the A-domain. These B-domain variants are known to interact with each other, promoting the formation of biofilms, especially in the presence of physiological concentrations of zinc (Geoghegan et al. 2010, Gruszka et al. 2012). While the A-domain is not thought to contribute to interbacterial attachment, it has been shown to bind to desquamated epithelium, invariably assisting in the process of biofilm formation (Roche et al. 2003, Geoghegan et al. 2010).

From studies with the comparatively better-characterized homolog Aap, it is clear that additional roles for SasG in *S. aureus* biofilm formation are likely. Studies by *Schaeffer et al*. (2015) demonstrate a significant reduction of biofilm burden in adult male Sprague-Dawley rats when jugular catheters were infected with a mutant unable to express Aap, in comparison to an isogenic wild-type control. Interestingly, this defect in virulence was only slightly increased when *Δaap* bacteria were unable to produce PIA, indicating a larger role for proteins, as compared to PIA, in assisting interbacterial attachment during biofilm formation. Indeed, SasG-expressing strains of *S. aureus* were found capable of forming PIA-independent biofilms. Lastly, in addition to potentiating interbacterial attachment, the fibrillar nature of SasG has been shown to mask the activity of other surface-associated proteins, including FnbpA and B. The role of SasG in facilitating biofilm development may therefore depend on the tissue niche as well as the presence of specific host proteins. Additional Sas family proteins include SasA (SraP), SasB, SasD, SasF, SasJ, SasK, SasL, and SasH (AdsA). Although the ligands for most of these proteins are currently poorly characterized, SasX and SasC have both been shown to be important for cell aggregation, likely promoting biofilm formation (Schroeder et al. 2009, Li et al. 2012).

*Immune evasion proteins*. While providing multiple avenues of adherence to the human tissue substratum, the surface-anchored proteins of *S. aureus* also contribute to pathogenicity and immune evasion mechanisms. The most versatile of these functions is performed by surface protein A (Spa), a cell surface-associated and secreted superantigen that is best known for binding to the Fc domains of IgG molecules, often masking their protective immune functions (Becker et al. 2014). Spa plays a particularly significant role in assisting the survival of *S. aureus* during bloodstream infections. This activity has specifically been correlated to an increased ability for recalcitrance in subsequent invasion of joint tissue, resulting in *S. aureus*-induced septic arthritis (Palmqvist et al. 2002). Furthermore, the immunoglobulin binding activity of Spa was utilized to demonstrate that Spa forms an essential component of polysaccharide-independent *S. aureus* biofilm matrices, since the addition of either serum, immunoglobulin, or anti-Spa antibodies showed a dose-dependent reduction of biofilm formation in a polysaccharide-independent manner (Merino et al. 2009).

As a prominent nasal colonizer, *S. aureus* expresses multiple surface proteins that facilitate bacterial binding to the nasal epithelium, some of which are described above. Additionally, the expression of a collagen-binding adhesin (Cna) provides the pathogen with specific access to the most abundant protein on the skin (Ricard-Blum 2011). Cna has been shown to additionally bind laminin, a commonly found basement membrane protein. It is therefore not surprising that Cna contributes to *S. aureus* colonization and the establishment of skin and implant-associated infections (Rhem et al. 2000, Arciola et al. 2005). In a study that genetically characterizes biofilm-forming *S. aureus* isolates from implant-associated infections, although MRSA strains were largely found to be Cna-negative, the adhesin was found to be expressed in ∼25% of implant-associated infections, indicating a specific role for the adhesin during biofilm formation in collagen-rich tissue environments (Arciola et al. 2005). As a consequence of its ability to bind collagen via its N-terminal domain, Cna has also been demonstrated to bind the collagen-rich triple helix stem domain of the serum complement activating protein, C1q (Kang et al. 2013). The stem of C1q is required for its activity. The Cna-C1q interaction therefore blocks complement activation, likely contributing to the evasion of innate immune defenses by *S. aureus* (Kang et al. 2013). Lastly, collagen has been reported to bind directly to fibrinogen and fibrin, a process that is crucial to wound healing (Hayuningtyas et al. 2021). The ability of *S. aureus* to bind collagen may therefore act as a bridge to accumulate fibrinogen around forming aggregates and mask the pathogen from immune defenses.

*The iron surface determinant (Isd) proteins*. As the only limiting nutrient for bacterial growth during infection, iron is required for bacterial survival in the human host. Conditions of iron starvation can alter the surface hydrophobicity of *S. aureus* (Clarke et al. 2004, Skaar et al. 2004). The iron sequestering, surface-anchored proteins of the Isd system, expressed via the *isdA, isdB, isdCDEFsrtBisdG, isdH*, and *isdI* transcriptional units, include IsdA, IsdB, IsdC, and IsdH. The promoters of these transcriptional units are repressed by the ferric uptake repressor protein (Fur) under iron-replete conditions. Studies by *Johnson et al*. (2005) describe an increase in biofilm formation under conditions of iron starvation. The authors note that this effect was independent of PIA production, suggesting a greater contribution for iron-scavenging proteins in the formation of PIA-independent matrix biofilms. Furthermore, the presence of hemoglobin in planktonic cultures has been associated with a downregulation of Agr, a process that occurs during the transition of planktonic populations to biofilm communities (Pynnonen et al. 2011).

In addition to their primary function, iron-scavenging proteins have demonstrated immune evasion properties that provide further evidence of their role during biofilm-associated infections (Clarke et al. 2007, Clarke and Foster 2008, Visai et al. 2009, Zapotoczna et al. 2013). *Torres et al*. (2006) clearly demonstrate that IsdB is essential for the binding of hemoglobin to the bacterial surface and that in the absence of this protein, *S. aureus* is severely impaired in its ability to cause kidney abscesses in murine models of infection (Kim et al. 2010). IsdA, while a structurally similar protein to IsdB, binds primarily to heme but has been shown to interact with additional ligands, including fibrinogen (Clarke et al. 2004). As a protein with a demonstrably significant contribution to nasal colonization, the expression of IsdA has been reported to reduce the hydrophobicity of the bacterial surface, consequentially making *S. aureus* tolerant to host fatty acids and immune cells as well as allowing for survival on human skin (Clarke et al. 2007).

#### Secreted proteins

In addition to a cache of secreted toxins and cytolytic enzymes used to lyse human cells, *S. aureus* expresses extracellular proteins that bind multiple host ligands, thereby facilitating aggregation and biofilm formation. The following section discusses these proteins and their roles in colonization and pathogenesis.

*Coagulases*. Survival in the bloodstream allows *S. aureus* to disseminate and cause life-threatening tissue-associated biofilm infections, including endocarditis, septic arthritis, and abscess formation. To interfere with blood homeostasis, *S. aureus* requires two coagulases, namely, staphylocoagulase (Coa) and vWf binding protein (VWbp), in conjunction with the fibrin binding capacity of ClfA. Indeed, the absence of any one of these secreted proteins results in a significant loss of virulence in bloodstream-associated infections by *S. aureus* (McAdow et al. 2011). Coa and vWbp are both mosaic proteins with N-terminal fibrinogen-binding domains. The N-terminus of both proteins also activates prothrombin. While the C-terminus of Coa acts as an additional fibrinogen-binding domain consisting of ∼27 tandem peptide repeats, the C-terminus of vWbp binds to fibronectin (Fig. 1A). The C-terminus of vWbp encodes a binding site for vWf. Since this host protein is present on endothelial tissue and vWbp is secreted, the significance of this interaction remains unclear. Recently, however, there has been much appreciation for the role of vWbp as a protein that bridges the bacterial surface to the hECM (Claes et al. 2014, Viela et al. 2019).

The activation of the tissue factor pathway at the site of injury is required for restoring homeostasis. The activity of multiple plasma proteases leads to the accumulation of Factor Va and Xa, also known as the prothrombinase complex, which activates prothrombin to form thrombin. The two secreted coagulases, Coa and vWbp can bypass these steps to catalyze the formation of enzymatically active staphylothrombin complexes. Similar to the activity of thrombin, staphylothrombin will cleave the fibrinopeptides A and B from fibrinogen to form fibrin cables (Fig. 1B). ClfA and FnbpA subsequently attach fibrin cables to the bacterial surface, thereby preventing the proper fibrin crosslinking that is required for clot formation under normal physiological conditions. This results in dense aggregate biofilms of bacteria shielded by a matrix of fibrin and bacterial proteins, protected from immune onslaught (Thammavongsa et al. 2013). Since these bacterial proteins circumvent the numerous enzymatic steps that are involved in physiological clot formation, this likely fails to induce the appropriate inflammatory response required to restore homeostasis (Thomer et al. 2016).

*DNA*-*binding proteins*. The characterization of polysaccharide- or PIA- independent biofilm formation by *S. aureus* has led to a greater appreciation for DNA and the proteins that comprise the EPS (Boles et al. 2010). The autolysin, Atl causes the enzymatic lysis of *S. aureus* growing as biofilms, resulting in a release of DNA that gets incorporated into the matrix (Bose et al. 2012). Additionally, cytoplasmic proteins “moonlight” as DNA-binding components when released into the matrix upon cell lysis. These proteins likely bind to DNA and protect it from the activity of nucleases released by the biofilm (Foulston et al. 2014, Moormeier et al. 2014). It is therefore evident that DNA is an important component of *S. aureus* biofilms. Additionally, *S. aureus* secretes a group of proteins with non-specific DNA-binding activity. Primarily characterized for their function in planktonic cultures, we recently identified these proteins from the matrix of PIA-independent biofilms using southwestern blotting and mass spectrometry (Kavanaugh et al. 2019). The most abundant of the proteins identified was the extracellular adherence protein, Eap. IsaB, a second, well-characterized cell wall-associated DNA-binding protein, was also identified in this screen. These two proteins play additive roles in retaining DNA onto the biofilm matrix (MacKey-Lawrence et al. 2009). Eap is additionally implicated as being able to bind and aggregate neutrophil DNA. *Eisenbeis et al*. (2018) utilize chemically induced neutrophil extracellular traps incubated with increasing concentrations of purified Eap to demonstrate a reduction in the induction of NETs *in vitro*. However, since NETosis is induced by *S. aureus* biofilms both *in vitro* and *in vivo*, the implications of this function require further research (Bhattacharya et al. 2018). Lastly, *Yonemoto et al*. (2019) find that SasG can compensate for the absence of Eap expression in an Eap-overexpressing strain of *S. aureus*. Their studies describe a role for Eap in maintaining the “ruggedness” of a biofilm, defined by the authors as a biofilm with decreased smoothness compared to a mutant unable to express SasG. Using gel shift assays with purified SasG, the authors conclude that SasG is also a DNA-binding protein and that Eap and SasG work to prevent the degradation of eDNA incorporated in the matrix. Whether this function contributes to the biofilm-associated phenotypes observed is unclear.

*Metabolic proteins. Foulston et al*. (2014) propose that *S. aureus* biofilm matrices are composed of cytoplasmic proteins released when bacteria enter into stationary phase, rather than the expression of specific matrix proteins when bacteria transition from planktonic to biofilm communities. The authors demonstrate that these intracellular proteins relocate to the cell surface in response to decreased pH and that this localization can be reversed with an increase in pH. Whether these proteins are actively translocated (secretion system) or released via autolysis, however, warrants further investigation. Biofilm metabolism is often found to have a complex but specific relationship with host responses. As an interesting example of this concept, *Tomlinson et al*. describe the process by which *S. aureus* glycolysis induces mitochondrial stress in the airway, resulting in the synthesis of itaconate, which re-directs *S. aureus* towards biofilm formation and away from glycolysis. In agreement with these observations, non-synonymous mutations in pro-glycolytic genes were discovered in isolates from chronic airway infections with *S. aureus*. These included mutations in pyruvate kinase (*pyk*), lactate dehydrogenase (*ldh*), and aconitase (*acnA*) (Heim et al. 2020, Tomlinson et al. 2021).

## Host extracellular matrix proteins that are exploited for biofilm formation

The study of microbial biofilms often focuses on the properties of the microbe. Similarly, research with host-biofilm relationships is often limited to the properties of immune cells. While this has greatly advanced the field of infectious disease control, there can be no effective treatment against *S. aureus* biofilm infections without a greater appreciation for the host-derived molecules that interact with and often assist the formation of *S. aureus* biofilms *in vivo*. In this section, we therefore focus on the general properties and known functions of some of the hECM molecules that are significant to *S. aureus* biofilm formation (Fig. 1B).

### Collagen

As the most abundant component of the extracellular matrix, each collagen molecule is composed of 3 polypeptide alpha chains with a characteristic Gly-X-X’ motif, where X is most often a proline and X’ is either a hydroxyproline or hydroxylysine (Fig. 1B). These α-chains assemble as a right-handed helical structure and this motif or collagen-like domain can be additionally found in numerous human proteins, including complement C1q, pulmonary surfactant proteins, and ficolins (Ricard-Blum 2011). Vertebrates express up to 46 distinct chains that can form 28 types of collagen and assemble as fibrils or networks, ubiquitous to the hECM in most tissues. While multiple hECM proteins contain collagenous subdomains, many hECM components, including fibrinogen and fibronectin can bind directly to collagen. This is of particular significance when thinking of *S. aureus* since the pathogen expresses proteins to bind collagen, fibrinogen as well as a fibronectin (Fig. 1A,B) (Bingham et al. 2008, Ganesh et al. 2008, O'Neill et al. 2008, Valotteau et al. 2017).

Vascular injury exposes underlying collagen, which binds to platelets with high affinity under shear conditions present in blood vessels (∼800 s^−1^) (Westerbacka et al. 2002). This interaction between collagen and platelets is additionally significant during the inflammatory stage of wound healing. Cytokines released in part due to the formation of fibrin clots will result in the influx of fibroblasts, epithelial, and endothelial cells. Fibroblasts can additionally deposit collagen at the wound site, contributing to wound closure. The breakdown of collagen, likely by matrix metalloproteases, results in the synthesis of growth factors and fibroblast proliferation, which further assist the processes of angiogenesis and re-epithelialization (Mathew-Steiner et al. 2021).

Some of the common manifestations of collagen overproduction include fibrosis, cirrhosis, and scar tissue. For example, patients born with mutations in the cystic fibrosis (CF) transmembrane conductance regulator are predisposed to the development of lung fibrosis. Studies with 3D-reconstructed CF airway stromal connective tissue demonstrate an increased production of collagen from CF-fibroblasts, in comparison to a non-CF control (Mazio et al. 2020). Similarly, alveolar tissue from CF patients was found to contain significantly higher levels of collagen as compared to age-matched healthy individuals, further underlining the central role of this hECM protein during disease (Ulrich et al. 2010).

### Fibronectin

Fibronectin is a ubiquitously distributed, multidomain, 440 kDa glycoprotein that plays a vital role in the cellular development of vertebrates and is subsequently involved in numerous homeostatic functions, including wound healing and blood clotting. Early research with this protein demonstrated that mouse embryonic development was severely impaired in the absence of fibronectin (Boucaut et al. 1984, George et al. 1993, Speziale et al. 2019). Fibronectin is found either as a soluble dimer in plasma (pFN1) or in its insoluble fibrillar form in tissue hECM (cFN1), where it plays various tissue-specific roles (Fig. 1B). Both types of fibronectin can self-associate, bind to cellular integrin receptors (primarily α5β1), and additionally to other hECM proteins, including but not limited to heparin, fibrin, collagen, tenascin, and fibrillin (Henderson et al. 2011, Spada et al. 2021). Cellular fibronectin is particularly well researched for its ability to carry out fibrillogenesis, a process that ultimately expands and provides architectural properties to the hECM. This is essential for the ability of cytokine, chemokine, and growth factor diffusion into tissue environments, thereby indirectly playing a major role in the host response to infection and disease (Spada et al. 2021). While adhesins of *S. aureus* are the best-characterized of the bacterial proteins that bind to fibronectin, both Gram-positive (*Streptococcus, Enterococcus, Clostridia*, and *Listeria* species) and Gram-negative (*Escherichia, Campylobacter, Salmonella* and *Borrelia*) bacterial pathogens have been shown to express proteins that can bind fibronectin (Speziale et al. 2019).

### Laminin

Laminin composition dictates the structure of basement membranes, a specialized hECM that is required for physically connecting all intra- and intercellular tissue environments. While laminins are a large group of glycoproteins that can vary in size and structure, they are primarily composed of three disulfide-linked polypeptides, namely the α, β, and γ chains (Fig. 1B). While the N-terminus of the glycoprotein is thought to be responsible for binding to other hECM components, including heparin and collagen, the C-terminus arguably performs the most important general function of binding to plasma membrane-anchored proteins of neighboring cells, thereby allowing communication between the intra- and extracellular compartments (Singh et al. 2012, Mckee et al. 2019).

Of note, the C-terminus of laminin is also where bacterial adhesins can bind, thereby potentially blocking essential intercellular biochemical signals from being transmitted, while utilizing laminin as a component of the biofilm matrix (Carneiro et al. 2004). Studies with mouse models of meningitis indicate that three major blood-brain barrier pathogens—*Neisseria meningitidis, Haemophilus influenzae*, and *Streptococcus pneumoniae*—can all manipulate the laminin binding receptor sites on brain endothelial cells, utilizing them as binding sites (Orihuela et al. 2009). These studies, as well as additional and similar investigations with *P. aeruginosa, Streptococcus agalacticae, Bacillus anthracis*, and *S. aureus* indicate a correlation between the ability to bind laminin and the potential for invasiveness of the pathogen (Carneiro et al. 2004, Tenenbaum et al. 2007, Wang et al. 2016, Paulsson et al. 2019).

### Elastin

Soluble tropoelastin monomers of ∼60 kDa multimerize to form elastin fibers. The monomer itself can extend up to eight times its own length (Uitto 1979, Swee et al. 1995, Baldock et al. 2011). As fibers, elastin is a highly hydrophobic molecule with a low elastic modulus that can resist acid and alkali attacks. As the name suggests, elastin is responsible for imparting elasticity to various cells, the most significant of these being the vascular tissue. Major blood vessels and aortas are composed of 25%–32% dry weight elastin (Uitto 1979, Ozsvar et al. 2021, Wang et al. 2021). As a consequence of its ubiquity in blood vessels, perturbations in elastin composition are associated with numerous cardiovascular conditions, including atherosclerosis, myocardial ischemia, reperfusion injury, and atrial fibrillation (Wang et al. 2021). The property to confer tissue elasticity is also of particular significance to the cells of the lung and female reproductive tract with elastin being abundant in the uterus and placental tissue (Ozsvar et al. 2021). Among the pathogens with documented ability to bind elastin to its advantage, *P. aeruginosa* is of particular note for expressing an elastase, pseudolysin, that can rapidly degrade the elastin present in lung tissue. Indeed, the significance of this singular interaction to the bacterium is evident in the fact that it is the most abundant extracellular virulence factor and protease that is expressed by clinically relevant strains of the pathogen (Hamdaoui et al. 1987). This elastase has also been demonstrated to play a significant role in degrading elastin fibers during bloodstream infections as well as corneal keratitis, further indicating its importance as a virulence factor, likely underlying some of the tissue preference observed with *P. aeruginosa* infections (Hamdaoui et al. 1987, Matsumoto 2004, Yang et al. 2015).

While binding to elastin has been observed with *S. aureus*, the consequences of this interaction to infection are currently underappreciated (Downer et al. 2002). Interestingly, similar to collagen, the concentrations and synthesis of elastin were also found to be higher in patients with cystic fibrosis and COPD, conditions that are often associated with biofilms of *P. aeruginosa* and *S. aureus. Mycobacterium tuberculosis*, the causative agent of tuberculosis, a devastating pulmonary disease, expresses three major secreted antigens, namely Ag 85A, Ag 85B, and Ag 85C, collectively known as the antigen 85 complex. In addition to playing significant roles during pathogenesis, these proteins were found to interact with tropoelastin and facilitate the binding of *M. tuberculosis*. Authors found that in the absence of tropoelastin binding ability, there was a significant reduction in the capacity of *M. tuberculosis* to invade cells (Kuo et al. 2013). These and similar studies provide further evidence of the central role that elastin can play during bacterial infection, warranting further investigations of its role in *S. aureus* biofilm structure and pathogenesis.

### Fibrinogen

Fibrinogen is a 340-kDa glycoprotein that polymerizes into fibrin and is essential for clotting and homeostasis. Fibrin was discovered as early as 1666 by Marcelo Malpighi in his seminal report titled “De polypo cordis” (Forrester 1995). The soluble fibrinogen monomer is primarily synthesized in hepatocytes and is a homodimer consisting of 2Aα, 2Bβ, and 2γ polypeptides linked by 29 disulfide bonds to form hexameric complexes (Fig. 1B). In healthy human subjects, soluble fibrinogen is present at 2–5 mg/ml but this concentration can go up to ∼7 mg/ml under acute inflammatory conditions. The polymerization of fibrinogen to fibrin requires the plasma-associated protease, thrombin to cleave fibrinopeptides from the N-terminus of Aα and Bβ polypeptide chains. Subsequently, the exposed “knobs” of these subunits are then inserted into “holes” in the β and γ chains of adjacent monomers. This results in the formation of a “protofibril.” These protofibrils will then aggregate and crosslink to form fibrin cables that form the network of a blood clot. As described in previous sections, *S. aureus* coagulases (Coa, vWbp) and ClfA can hijack this homeostatic process to form “thrombi” containing bacteria shielded by fibrin, a form of biofilm formation that is characteristic to *S. aureus* (Thomer et al. 2016).

The structure and stability of fibrin clots can depend on integral blood properties, including but not limited to the concentration of anticoagulants, pH, temperature, blood flow, platelet, and red blood cell compositions. Lastly, perturbations in the structure and concentration of fibrinogen itself can alter the physical properties of fibrin and its subsequent effectiveness at restoring hemostasis. These changes can occur due to multiple pre-disposing conditions, including but not limited to ischemic stroke, myocardial infarction, aortic aneurysms, and thromboembolisms (Kattula et al. 2017, Pieters and Wolberg 2019).

## The role of matrix proteins during *S. aureus* biofilm infection—a few key examples (see Fig. 2 for graphical summary)

### Skin infections

Human skin forms the outermost barrier that protects the body against pathogens. Some of the innate properties of skin that prevent bacterial proliferation include low pH, sebaceous gland secretions, antimicrobial peptides, and lipids. Normal flora can colonize every major layer of the skin, including the epidermis, dermis, subcutaneous, adipose, and muscle fascia. These populations often provide an additional barrier against pathogens by outcompeting foreign species. *Staphylococcus epidermidis* is a commensal on healthy skin and is the most prominent antagonist for the development of *S. aureus* biofilms (Lunjani et al. 2019).

Specifically, *S. epidermidis* secretes a protease (Esp) that can reduce the ability of *S. aureus* biofilm formation. Indeed, nasal swabs obtained from patients with Esp-secreting *S. epidermidis* were associated with the absence of *S. aureus*, indicating that this commensal can actively prevent *S. aureus* nasal colonization (Dubin et al. 2001, Vandecandelaere et al. 2014). Upon perturbation of the normal flora or when allowed to gain entry via abrasions or injections, *S. aureus* invariably causes devastating skin infections that can lead to chronic biofilm-associated conditions, the most common of which are discussed below.

#### Atopic dermatitis

Atopic dermatitis is a chronic inflammatory skin disease commonly associated with *S. aureus* (39%–70%) and prominent in children (15%–20%) (Park et al. 2013, Totté et al. 2016). AD patients with large numbers of *S. aureus* often correlate with lower densities of the commensal, *S. epidermidis*. This pattern is also associated with severe cases of AD. Also of particular interest is the association of *S. aureus* biofilm-forming propensity with severe AD cases. *Gonzalez et al*. (2021) used 400 AD isolates of *S. aureus* to show that 62% had high to moderate biofilm-forming capacity. Additionally, although 68% of those that were co-colonized with *S. epidermidis* and *S. aureus* formed mixed species biofilms, severe AD was only associated with strong biofilm-forming isolates of *S. aureus* and not *S. epidermidis*. In another study performed with *S. aureus* isolates from patients with AD, *Cho et al*. (2001) demonstrate that strains expressing collagen-binding adhesin as well as fibronectin-binding proteins were better able to adhere to stratum corneum isolated from AD patients as compared to healthy skin as well as bacteria that could not express adhesins required to bind to either collagen (Cna) or fibronectin (Fnbps). Two additional cell wall-associated proteins, ClfB and FnbpB, have specifically been implicated in this increased binding to AD stratum corneum. Additionally, *Towell et al*. (2021) elegantly demonstrate the direct binding of ClfB and FnbpB to corneodesmosin, the major protein found in corneocytes on the skin. Of note, the stratum corneum of AD patients contains markedly lower levels of natural moisturizing factor, which leads to an abnormal level of exposed corneodesmosin, likely allowing for *S. aureus* proteins to bind and potentiate biofilm formation. These studies indicate the significance of hECM-binding proteins as well as biofilm formation, to the pathogenesis of *S. aureus* AD infections.

*Chronic Wounds*. A wound that is physiologically impaired due to a disruption in the wound healing cycle, often resulting in a prolonged inflammatory phase, is defined as a chronic wound. These include diabetic ulcers, venous ulcers, and pressure ulcers (Bjarnsholt et al. 2008, Percival et al. 2012). There are four main stages of wound healing namely hemostasis, inflammation, proliferation, and remodeling. Clotting and platelet plug formation are necessary to achieve hemostasis. Specifically, platelets bind to collagen exposed in the endothelial wall as well as fibrinogen and fibrin, resulting in the development of a plug (Wilkinson and Hardman 2020). This process can be interrupted by multiple *S. aureus* proteins, as described in previous sections. The inflammatory phase of wound healing begins with the recruitment of neutrophils as a response to platelet signals. Neutrophils use phagocytosis, degranulation, and NETosis to eliminate the infection, but often fail (Schierle et al. 2009, Roy et al. 2014, Jeffery Marano et al. 2015, Wilkinson and Hardman 2020). This is in part due to a variety of exotoxins and immune evasion factors expressed by the pathogen. *Staphylococcus aureus* biofilms in particular are recalcitrant to neutrophil defense mechanisms, expressing leucocidins that induce the release of NETs, which fail to clear the biofilm (Bhattacharya et al. 2018, 2020). Simultaneous to the neutrophil response, monocytes are recruited to the site of infection by hECM proteins, including collagen, fibronectin, serum complement factors, and elastin. Monocytes will differentiate into macrophages and assist the removal of neutrophil and bacterial debris generated in the process. The last cells to be recruited are lymphocytes that respond to similar cues, including complement, degraded immunoglobulin, and cytokines. Resolution of the inflammatory phase involves the replenishment of some hECM components such as collagen, by resident fibroblasts (Wilkinson and Hardman 2020). Proliferation, also known as granulation tissue formation, includes the development of new connective tissue with rapid and extensive replenishment of hECM proteins characterized by the replacement of the fibronectin matrix by a stronger, collagen I and collagen III-rich scaffold (Wilkinson and Hardman 2020, Mathew-Steiner et al. 2021).

Multiple studies demonstrate that *S. aureus* is one of the most commonly isolated pathogens in both acute (∼60%) and chronic wound infections (∼65%) (Gjødsbøl et al. 2006, Kirketerp-Møller et al. 2008, Fazli et al. 2009, Percival et al. 2010). Other pathogens frequently isolated include *P. aeruginosa* and *Enterococcus* species, both prominent biofilm-forming bacteria. Indeed, a growing body of literature demonstrating the presence of biofilms in these wounds indicates a need to consider biofilm development as one of the hallmarks of chronic wound infection by bacterial pathogens (Gjødsbøl et al. 2006, Bjarnsholt et al. 2008, Kirketerp-Møller et al. 2008, Fazli et al. 2009). Studies with both acute and chronic models of wound infections indicate that *S. aureus* biofilms delay wound healing by causing inflammation and delayed re-epithelialization (Schierle et al. 2009, Chaney et al. 2017). A number of infection-related phenotypes are associated with responses generated through the host extracellular matrix. Of note, chronic wounds infected with *S. aureus* cause the activation of tissue- and neutrophil-derived metalloproteases, which breakdown collagen. Seminal studies in the field of biofilm-associated wound infections describe a role for matrix metalloproteinase-2 in reducing the ratio of collagen1/collagen 3 present at the wound edge, resulting in a loss of skin tensile strength (Roy et al. 2020). Results from this study support the recurrence of *S. aureus* biofilms in these poorly re-epithelialized wounds. Other groups have also shown a correlation between the presence of Staphylococci in wounds and an increase in epithelial gap size (Xu et al. 2021).

### Lung infections

While the cellular composition of the human lung is incredibly complex and yet to be fully understood, it can largely be divided into the parenchyma and the interstitial space. A characteristic feature of the lung epithelial barrier is the presence of a ciliated pseudostratified columnar epithelium coated with mucus that is produced by goblet cells found throughout the airway. This is arguably the most important defense against those pathogens that can gain entry to the respiratory system through the nose and mouth (Burgstaller et al. 2017, Hall-Stoodley and McCoy 2022). Proteomic studies reveal that the pulmonary interstitial space can contain at least 150 hECM components, with perturbations in the levels of these constituents often resulting in deleterious effects such as heightened disease progression (Shimbori et al. 2013, Eurlings et al. 2014). For example, researchers have found that the regeneration of lung matrix components by resident fibroblasts plays an important role in maintaining homeostasis after injury or infection (Sivakumar et al. 2012, Shimbori et al. 2013).

Regardless of location, collagen is the most abundant protein in the lung. Since the lung is a diverse microenvironment, the composition of hECM as well as collagen subtypes observed differs depending upon whether tensile strength or elasticity take functional preference (Burgstaller et al. 2017, Hall-Stoodley and McCoy 2022). For example, the alveoli, responsible for gas exchange, are provided with an elastic hECM matrix composed mainly of collagen I and III as well as elastin, which allows this environment to maintain non-linear stress-strain behaviors that are characteristic of connective tissue. Another important hECM component in the alveolus is the negatively charged proteoglycan heparin sulfate, which interacts with numerous bioactive compounds and is often shed into the alveolar space for up to 3 weeks post-insult. This can result in detrimental effects to the host, including inflammation and decreased barrier function (Haeger et al. 2018, LaRivière et al. 2020). The overall mechanical integrity of the lung is largely provided by the fibrillar, less elastic collagen types I, II, III, V, and IX. In addition to collagen and elastin, lung hECM also consists of glycosaminoglycans, fibronectin, laminin, heparin sulfate, and proteoglycans such as decorin and biglycan among others.

Although the structural complexity of the lung impedes the ease of demonstrating biofilm-associated infections in this environment, recent advances in confocal microscopy and 16s RNA sequencing have provided clear evidence of the presence of these communities. For example, *Kolpen et al*. (2022) collected sputum samples from 43 patients (16 pneumonia, 13 COPD, and 14 CF) and showed that biofilm communities of >100 um^3^ could be observed in 40 of these samples. These studies provide evidence for the presence of biofilms in both acute and chronic airway infections. Some key examples of these biofilm-associated infections are provided here.

*Pneumonia*. Bacteria and viruses are common causes of infection in the lung. This pneumonia is often characterized by inflammation of the lung tissue and accumulation of fluid or pus in alveoli. Two of the most common types of pneumonia associated with *S. aureus* infections include hospital-acquired pneumonia and ventilator-associated pneumonia (VAP). VAP is caused in patients that require mechanical ventilation following tracheal intubation, often utilized as a method to alleviate breathing distress from previous conditions such as emphysema, heart failure, or severe trauma (Becker and Kerr 1960, Parker and Prince 2012, Pickens and Wunderink 2022). These infections are most common in patients with compromised immunity such as infants (<5 yrs) and elderly patients (>65 yrs) as well as patients undergoing prolonged treatment (Risk Factors for Pneumonia | CDC 2022; Parker and Prince 2012). Colonization of the nasal epithelium is a prerequisite to the development of lung infection. Two surface adhesins, namely clumping factor B (ClfB) and collagen-binding adhesin (Cna) have been found to be associated with efficient colonization. While ClfB is required for attachment to cytokeratin in the nasal epithelium, Cna was found to be important in bacterial attachment to collagen I and IV as well as laminin, prominent in the basement membrane tissue. The expression of Cna is known to be a predisposing factor for the ability of *S. aureus* to cause necrotizing pneumonia (De Bentzmann et al. 2004). It is likely that since *S. aureus* colonizes the anterior nares of ∼30% of the population, pneumonia can arise as a secondary infection, caused by multiple primary insults. *Staphylococcus aureus* is commonly acquired as a secondary, severe infection following influenza. Recent studies by *Langouët*-*Astrié et al*. (2022) demonstrate that the damage caused to lung tissue, specifically the alveolar lining, following an influenza infection, results in excessive shedding of heparin sulfate, a prominent pulmonary glucosaminoglycan. This causes a significant increase in the expression of cytotoxins by *S. aureus*, often resulting in fatality due to secondary bacterial infection (Teng et al. 2019). Interestingly, research with mixed biofilms of *S. aureus* and *S. pneumoniae*, another prolific cause of community-acquired pneumonia, demonstrates that when mice colonized with both bacteria are challenged with a secondary influenza virus infection, *S. pneumonia* biofilms can disperse, while preventing the dispersal and therefore the transition to invasive disease of *S. aureus* (Reddinger et al. 2018).

Although much remains to be understood about the pathogenicity of biofilms in the lung, the development of tools required to demonstrate the presence of these communities during pneumonia infection is rapidly growing. *Baidya et al*. (2021) analyzed bronchoalveolar lavage and deep aspirate from 70 patients with VAP to show that biofilms could be observed in 56.3% of these samples. Additionally, studies by *Hook et al*. make direct confocal observations of USA300 *S. aureus* labeled with green fluorescent protein and show that following inhalation of these bacteria by mice, *S. aureus* travels to the lungs, where they form microaggregates in >50% of the alveoli observed. These communities were found to be stable and could not be flushed out with buffers. Furthermore, these microaggregates express cytotoxins, caused inflammation and localized lung injury while being impermeable to antibiotic-sized solutes (Hook et al. 2018). Together, these results emphasize a need for the consideration of biofilms as a common phenotype in bacterial pneumonia.

*Cystic Fibrosis*. A monogenic recessive mutation in the cystic fibrosis transmembrane receptor (CFTR) causes impaired mucociliary clearance and increased susceptibility to numerous pathogens, including but not limited to *P. aeruginosa, S. aureus, Haemophilus influenzae, Burkholderia cepacia*, and *Mycobacterium abscesses* (Harrison 2007, Hall-Stoodley and McCoy 2022). The CFTR is responsible for the transport of chloride and bicarbonate across the epithelial lining. This mutation, therefore, has widespread effects, including a disruption of pancreatic and gastrointestinal function, in addition to its most prominent manifestations in the lung (Wallis 1997, Cohen and Prince 2012). The most common consequence of CFTR mutations is the excessive uptake of water by epithelial cells, which leads to a concomitant depletion of fluidity on the airway surface fluid and thickening of mucus. Mucociliary clearance is hampered, allowing for pathogens to be retained and infections to develop with much greater ease than they would in a healthy lung (Gilligan 1991, Hauser 2009, Zemanick and Hoffman 2016). The lung of a CF patient is a uniquely harsh environment for the growth of microbes. Increased acidity, oxidative stress, hyperinflammation, and competition between multiple microbial species result in the selection of microbial sub-populations that adapt to withstand these conditions. This includes the development of mucoidy, small colony variants, and biofilms (Pietruczuk-Padzik et al. 2010, Høiby et al. 2017).

As a result of numerous insults to the lung, tissue scarring or fibrosis causes difficulty in breathing. The process of fibrosis in and of itself is defined by the overproduction of hECM (Henderson et al. 2020). *Staphylococcus aureus* is one of the first bacteria detected in infants and children with CF (Harrison 2007). Recent patient registry information from Europe and the USA shows that 60%–80% of CF patients under the age of 20 are colonized with *S. aureus* (ECFS Patient Registry Annual Data Report 2020, Patient Registry | Cystic Fibrosis Foundation 2021). As a common resident of the anterior nares, *S. aureus* finds easy access to the respiratory tract of this immunocompromised population. Additionally, with frequent hospital stays, the introduction of *S. aureus* biofilms via biotic surfaces such as syringes, catheters, and endotracheal tubes as well as through surgical procedures and contact with healthcare workers is common (Saiman et al. 2014, Savant et al. 2014, Bell et al. 2018).

Patients with CF develop hyperinflammation months after birth. In combination with repeated microbial infections, the cycle of inflammation and tissue destruction perpetuates over their lifetimes (Cantin et al. 2015). Neutrophil-induced inflammation is a pathophysiological hallmark of CF disease and results in the release of numerous proteolytic factors, including matrix metalloproteases, Cathepsin G, and neutrophil elastase that destroy the connective barrier of the lung, reducing the capacity of tissue to expand and contract while breathing. Interestingly, Cathepsin G can effectively disrupt *S. aureus* biofilm i*n vitro*. Whether this activity can clear biofilms in the lung environment, however, remains to be investigated (Kavanaugh et al. 2021).

Cross-sectional biochemical analyses performed on bronchioalveolar lavage fluid from children with CF describe an extensive remodeling of the lung hECM with a dramatic increase in the levels of elastin, collagen, and glycosaminoglycans. Early work in the field describes an increased concentration of collagen-derived peptides and elastin degradation products (desmosins) measured in urine collected from CF patients, when compared to healthy individuals (Stone et al. 1995). Since then, studies with CF sputum samples show significantly elevated levels of collagenase, specifically derived from neutrophils (Power et al. 1994). Recently, *Pinezich et al*. (2022) conducted an elegant mass spectrometric analysis of explanted lungs from end-stage CF patients in comparison to non-CF transplant donors to characterize the major changes that occur in the matrix of these tissues. Their results showed that there is a significant degree of degradation in the extracellular matrix structure and composition in CF patients, when compared to non-injured donors. Similar pathology is observed in a less common form of fibrosis, namely idiopathic pulmonary fibrosis. Increased deposition of collagen is often reported, with decline in neutrophil function and a concomitant prevalence of *S. aureus* infections (Warheit-Niemi et al. 2020). Together, these studies indicate that hECM disbalance occurs and significantly contributes to the pathology during CF. As a majority of the research in the field of CF focuses on *P. aeruginosa*, the most common biofilm-forming pathogen infecting adults, much remains to be understood about the consequence of these hECM perturbations to the development of *S. aureus* biofilm infections, prevalent in children with CF.

### Device-related infections

Hospital-acquired infections often arise from contaminated medical and prosthetic devices. The most significant number of these cases are caused by *S. aureus* and *S. epidermidis* (Stoodley et al. 2013). Due to its ability to rapidly acquire tolerance and resistance to numerous antibiotics, *S. aureus* is a particularly problematic cause of foreign body-associated infections. Devices most commonly associated with *S. aureus* infections include catheters (central venous and urinary), heart valves, pacemakers, endotracheal tubes, contact lenses, and periprosthetic devices (Moran et al. 2010, Peel et al. 2011). The most likely mechanism for entry of the pathogen and colonization on these devices is during surgical procedures or during the insertion of the device in patients that are colonized with *S. aureus* (McConoughey et al. 2014). Infection in the device can often spread to neighboring soft tissue and bone and may result in sepsis, especially when the device is inserted into the heart or circulatory system (Manne et al. 2012, Osmon et al. 2013).

Once an implant has been inserted into the body, it is immediately covered in plasma proteins, including vWf, fibrinogen, fibronectin, and vitronectin. This process is crucial in allowing the implant to be accepted as “self” by the host immune system. Unfortunately, this coating provides for a “conditioned” surface that is perfect for bacteria to attach to, proliferate on, and form biofilms. This environment is therefore particularly favorable for biofilm formation by *S. aureus*, owing to its ability to bind each of these hECM components. *Pestrak et al*. (2020) demonstrate the nature of this double-edged sword in that while synovial fluid surrounding periprosthetic joints prevents bacterial attachment to the implant surface, fibrin and fibrinogen present in the fluid can promote the formation of bacterial aggregate biofilms that survive in synovial fluid to potentially cause secondary infections. As described by these studies, the fibronectin-binding protein A (FnbpA) is of particular relevance to implant-associated infections. FnbpA is a surface-associated protein that was found to be encoded in ∼99% of strains isolated from 191 implant-associated infections (Arciola et al. 2005). In a rodent model of implant infection, *Gries et al*. (2020) demonstrate that the expression of FnbpA allows for *S. aureus* to form structured communities on Teflon-coated intravenous catheters and prevents bacterial clearance by macrophages. Additionally, the authors describe a role for this protein in allowing for *S. aureus* to spread to neighboring soft and bone tissue.

The fibrinogen-binding ability of *S. aureus* can change the course of a bacterial infection to the detriment of the host. While this is discussed predominantly in the context of the bloodstream, it is extremely relevant to device-related infections by the pathogen. Seminal studies by *Vanassche et al*. utilized dabigatran, a pharmacological inhibitor of staphylothrombin formation, to show that treatment with this drug could significantly reduce the capacity of *S. aureus* to form biofilms on jugular vein catheters in a mouse model of infection. This was accompanied with a reduction in fibrin deposition and increased efficacy of vancomycin treatment (Vanassche et al. 2013). The conditioning of implant material is therefore a critical consideration that is often absent from *in vitro* analyses aiming to understand the pathogenesis of *S. aureus* biofilms during device-related infections.

### Endocarditis

Endocarditis is the inflammation of the endocardium, the inner squamous epithelial lining of the heart chambers, which connects the heart to the circulatory system through blood vessels. An infection of this lining termed infective endocarditis (IE), most often proves fatal. As early as 1973, *Durack et al*. (1973) used a rabbit model of endocarditis to demonstrate that the endocardium is recalcitrant to bacterial colonization. The development of an infection in this tissue therefore requires perturbations in the valvular surface that create a favorable environment for such infections. This often occurs as a result of injury, surgical intervention, or the introduction of foreign bodies, including pacemakers and heart valves. Other major factors that facilitate the development of endocardial biofilms or “vegetations” include the development of bacteremia caused by a primary infectious focal point such as an infected catheter or skin wound (Mylonakis and Calderwood 2001, Werdan et al. 2014). As the causative agent of 15%–40% of IE in Europe and the USA, mortality rates for cases caused by *S. aureus* are 20%–30% (Asgeirsson et al. 2018).

An increasingly problematic cause of IE is the intravenous use of drugs that provide direct access for *S. aureus* colonizing the skin or unhygienic abiotic surfaces into the bloodstream (Wang et al. 2023). One such case reported by *Rali et al*. (2019) at the University of Kansas Medical Center provides a comprehensive example for the transmission, manifestation, and treatment of IE. In this study, a 56-yr-old male was previously hospitalized due to the development of chronic purulent bilateral cellulitis and osteomyelitis caused by *S. aureus*. These infections were traced back to repeated intravenous injections in the lower extremities. As a testament to the difficulty in preventing biofilm-associated infections, especially IE, upon admission, while a transthoracic echocardiogram (TTE) was negative for IE, *S. aureus* was positively cultured from the blood and urine of the patient. Soon after presentation, this patient was admitted to the intensive care unit, where a subsequent TTE demonstrated the presence of a ∼0.5 cm vegetation. This was accompanied by vegetations in the mitral, tricuspid, and aortal valves, which progressed to the lungs and brain, proving fatal. This is one of many cases where the short time frame of biofilm development combined with the inability to make early detections in heart tissue, leads to devastating consequences, further emphasizing the need to acknowledge biofilms as a serious, unique infectious disease hurdle (Guler et al. 2013, Wang et al. 2023). The modified Duke’s criteria, widely used by clinicians to diagnose endocarditis are only useful once blood cultures turn positive for the presence of a particular pathogen, thus catching the infection long after biofilm formation would have occurred (Li et al. 2000).

When considering the extracellular matrix of the heart, it is important to note that the regenerative capacity of this organ is poor. This means that injury to cardiac tissue and muscle results in very low levels of replacement with healthy cells, as is the case during myocardial infarction (Bergmann and Jovinge 2014, Silva et al. 2020). The hECM in and around the endocardium is rich in collagens I, III, and IV, with types I and III being responsible for maintaining the mechanical strength of the compartments. Additionally, the major constituents of the hECM include fibronectin, elastin, laminin, proteoglycans, and glycosaminoglycans (Silva et al. 2020). In part due to the challenges of studying heart infections, especially *in vitro*, there is relatively little known about the pathogenesis of *S. aureus*-induced IE. In a well-designed attempt to improve some of these techniques, *Liesenborghs et al*. (2019) describe the development of a murine model for the study of endocarditis. By comparing the effects of inflammation and valvular damage, the authors are able to image the infiltration of granulocytes and platelets to the site of *S. aureus* biofilm formation. Using these methods, the authors were able to provide *in vivo* evidence for some key events that occur during IE with *S. aureus*. They show that valve damage and inflammation can cause differential immune responses to ensue. While both processes involved vWf, inflammation resulted in a predominantly platelet-driven mechanism of trapping the bacteria, independent of bacterial cell wall-anchored proteins. Conversely, valvular damage was accompanied by *S. aureus* binding directly to vWF (via the vWbp coagulase) and fibrinogen/fibrin via ClfA, which deposited onto the damage valve. This is one of very few examples of an *in vivo* exploration of the pathogenesis of *S. aureus* IE and brings up an important and underestimated aspect of *S. aureus* infection, namely the role of platelets (Niemann et al. 2004, Ali et al. 2017). A significant impediment in the ability of a bacterium to cause bacteremia and therefore IE is the requirement to attach to valvular surfaces in the presence of extreme shear stress generated by the flow of blood. While most IE pathogens bind to platelets in order to gain entry*, S. aureus* uses vWbp to mimic the mechanisms used by platelets binding to vWf that are exposed on the endothelial lining of damaged valves (Claes et al. 2014).

## Current therapeutics and future prospects

One mechanism for preventing the formation of an *S. aureus* biofilm *in vivo* is to block the activity of adhesins that bind hECM components. Currently, there is no approved vaccine available to prevent *S. aureus* infections, in part due to its ability to use multiple virulence strategies against the host. Numerous studies have attempted to develop vaccines that target the hECM binding domains of surface proteins, to varying degrees of success. An additional hurdle is the selection of a vaccine target that is conserved among strains of *S. aureus*.

*Mazmanian and Schneewind* demonstrated that Sortase A is essential for the cell wall anchoring of 23 surface-associated adhesins (Mazmanian et al. 1999). Further studies show that *ΔsrtA* sortase mutants of *S. aureus* cannot colonize the nasal passages or cause abscess formation in a well-established murine model of bacterial sepsis (Mazmanian et al. 2000, Cheng et al. 2009). Owing to its importance during infection, multiple efforts have been made to develop a multivalent vaccine that targets multiple sortase-anchored proteins. *Stranger-Jones et al*. (2006) performed one of the most comprehensive studies to this effect by generating antibodies against 19 highly conserved sortase-anchored proteins. Protection studies with mice showed that the most protective antibodies were generated against IsdA, IsdB, SdrD, and SdrE. The V710 vaccine was developed against IsdB and reached phase 3 clinical trials performed on patients undergoing cardiothoracic surgery. Administering V710 was not only ineffective at preventing infection but caused a higher degree of mortality in patients who developed *S. aureus* infections. While the reasons for this safety failure were not clear, it provides an insightful example of the complexity in pathogenesis and the need for the development of tissue-specific interventions (Fowler et al. 2013). There is an ongoing effort to discover inhibitors of Sortase A activity. The disadvantage of these compounds to clinical use, however, is that they are often not bactericidal and are therefore unlikely to eradicate the infection (Schneewind and Missiakas 2019).

Coating periprosthetic devices with anti-biofilm compounds is a burgeoning field of research for the prevention of the attachment phase of biofilm formation. Most of these efforts have focused on antimicrobial compounds and taken advantage of the hydrophobic nature of bacteria by creating hydrophilic and therefore repellant implant surfaces (Busscher and van der Mei 2012, McConoughey et al. 2014). While these methods are useful in the prevention of bacterial adhesion to surfaces, they do not alter the ability of bacteria to form aggregates with plasma-derived matrices. Consequently, anti-fibrinolytic agents such as dabigatran, plasmin, and warfarin are clinically utilized and can break up *S. aureus* aggregates that form with fibrinogen, fibrin, and fibronectin. *Hogan et al*. (2018) used a combination of antimicrobial and anti-fibrinolytic agents to demonstrate a significant reduction of bacterial growth in a rat model of catheter-associated biofilms. Similarly, *Kwiecinski et al*. (2016) show that coating polyester-based coverslips with tissue plasminogen activator, required for fibrin cleavage, was effective at reducing biofilm formation *in vivo* and improving the efficacy of clinically prescribed anti-*S. aureus* drugs. These studies demonstrate the need to target more than one biofilm properties in order to eradicate the infection.

A major issue with the use of agents that target bacterial-hECM interactions, is the requirement for localized delivery. While these methods are effective for implant-associated biofilms, the administration of antithrombotic agents can cause disastrous effects if delivered systemically. Additionally, the localized delivery of antibiotics would be more effective than systemic delivery routes (Howlin et al. 2015). Lastly, the formation of IE vegetation biofilms occurs similarly to blood clots and requires the activation of platelets. While a combination of anti-platelet and anti-thrombotic agents has successfully been utilized to show increased survival of rats in an *in vivo* model of IE, results with clinical trials show mixed results, with one study showing increased potential for bleeding when aspirin was used as an anti-platelet compound (Chan et al. 2003, Anavekar et al. 2007, Veloso et al. 2015). These studies, while unsuccessful to varying degrees, provide additional pieces to the puzzle of treating a biofilm infection and clearly define the essential role of ECM components to the survival of *S. aureus* biofilms *in vivo*.

## Conclusions

The effective treatment of *S. aureus*-associated infections requires a greater appreciation of biofilms as the predominant bacterial lifestyle (Costerton et al. 1978, Costerton et al. 1999, Donlan and Costerton 2002, Hall-Stoodley et al. 2004, Høiby et al. 2011). Concentrations of antibiotics used against planktonic *S. aureus* populations will therefore not be effective against biofilms and can lead to the development of extremely recalcitrant subpopulations, perpetuating the problems of antibiotic tolerance and resistance (Stewart and William Costerton 2001, Donlan and Costerton 2002). An additional hurdle that comes with the study and treatment of *S. aureus* biofilms, lies in the pathogenic versatility of the bacterium, expressing a diverse combination of virulence factors, the permutations, and combinations of which are highly complex and under tight temporal control. Therapeutics to eradicate this pathogen will therefore require a multi-target approach (Bhattacharya et al. 2015). Unlike most pathogens, *S. aureus* is uniquely designed to thrive in the human body during both colonization and infection. This is in part due to the expression of one or more surface or secreted bacterial components (Fig. 1A) with specific domains that form strong physical interactions with host extracellular ligands (Fig. 1B) (Foster 1998, Schneewind and Missiakas 2019). The ability to eradicate *S. aureus* biofilms is therefore intricately linked to the framework of the host itself and requires a deeper understanding of the biochemistry and functionality of various host proteins, carbohydrates, and lipids that actively interact with *S. aureus* to potentiate the formation and/or survival of biofilms *in vivo* (Deadly Staph Infections Still Threaten the USA | CDC Online Newsroom | CDC 2019; Bhattacharya et al. 2015). This review provides a comprehensive discussion of human extracellular proteins that are particularly relevant to the pathogenesis of *S. aureus*. As evident from the summary provided in Fig. 2, the complexity of these interactions can be highly dynamic and dependent on the tissue-specific abundance of each host protein. Dispersion of biofilm communities and the transition to more invasive bacterial virulence mechanisms are additional considerations that need to be recognized in order for research on *S. aureus* pathogenesis to reach greater clinical relevance.
